# A rare case and review of pulmonary ossification

**DOI:** 10.1016/j.rmcr.2022.101760

**Published:** 2022-11-04

**Authors:** Alexander J. Sweidan, Justin H. Shiu, Mohammed Y. El Naggar, Bahman Saatian

**Affiliations:** a Department of Medicine, Veteran Affairs Long Beach Healthcare System, Long Beach, CA, USA; b Department of Medicine, University of California, Irvine, CA, USA

**Keywords:** Pulmonary ossification, Pulmonary calcification, Dendriform pulmonary ossification, Nodular pulmonary ossification

## Abstract

Pulmonary ossification (PO) is a rare metastatic disease characterized by the formation of diffuse heterotopic bone units in the lung parenchyma. Herein, we describe a 45-year-old Filipino male with dendriform pulmonary ossification in combination with gastroesophageal reflux disease and chronic dust exposure, a notably unique association. Radiographic imaging and pathology findings are examined with discussion of various pulmonary disease entities from current literature. Further recognition of PO will facilitate appropriate treatment and better outcomes for patients diagnosed with this enigmatic condition.

## 1 Introduction

Pulmonary ossification (PO) is a rare disease entity that is often identified radiographically in asymptomatic middle-aged men [1,2]. PO is bone formation (calcification in a collagen matrix), with or without marrow elements, in the lung [3]. While other organs can be affected by ectopic ossification, the lungs are especially vulnerable [3]. Pulmonary calcifications that occur without bone formation is typically identified as metastatic, dystrophic or as pulmonary alveolar microlithiasis [3]. Pulmonary calcification without bone formation is beyond the breath of this review. Herein we describe a case of PO followed by a thorough discussion on the various sub entities.

## 2 Description

A healthy 45-year-old Filipino male initially presented to the emergency department with flu-like symptoms and cough. He also endorsed rare chest burning after heavy meals which was managed with over-the-counter antacids as needed. Prior to these symptoms, the patient had excellent exertional capacity such as playing sports with his children and running and climbing multiple flights of stairs without respiratory ailment.

A history relevant for gastroesophageal reflux disease was recorded. Otherwise, the patient denied any chronic lung conditions such as asthma, recurrent respiratory infections, or pneumonia. No outstanding surgical history. Travel history was unremarkable.

He did transiently smoke cigarettes for 9 months, approximately half a pack a day when he was in his early 20s. He works as an administrator for an office space that renders him at his desk most of the day. He denied exposure to fumes, gas chemicals, or hard metals. He is a Veteran and served in the Gulf War for 7 months. Thereafter, he spent another 8 months in Iraq working in a dusty warehouse for out-processing mail. He never owned any pets. He denied sleeping on or using down material.

Vital signs were stable on initial presentation: afebrile, resting pulse oximetry of 99% with heart rate of 77 beats per minute, blood pressure of 133/76 mm of mercury. Body mass index was noted to be 26. His lungs were clear to auscultation without any wheeze or crackles. All other physical exam findings were normal.

**Fig. 1:**
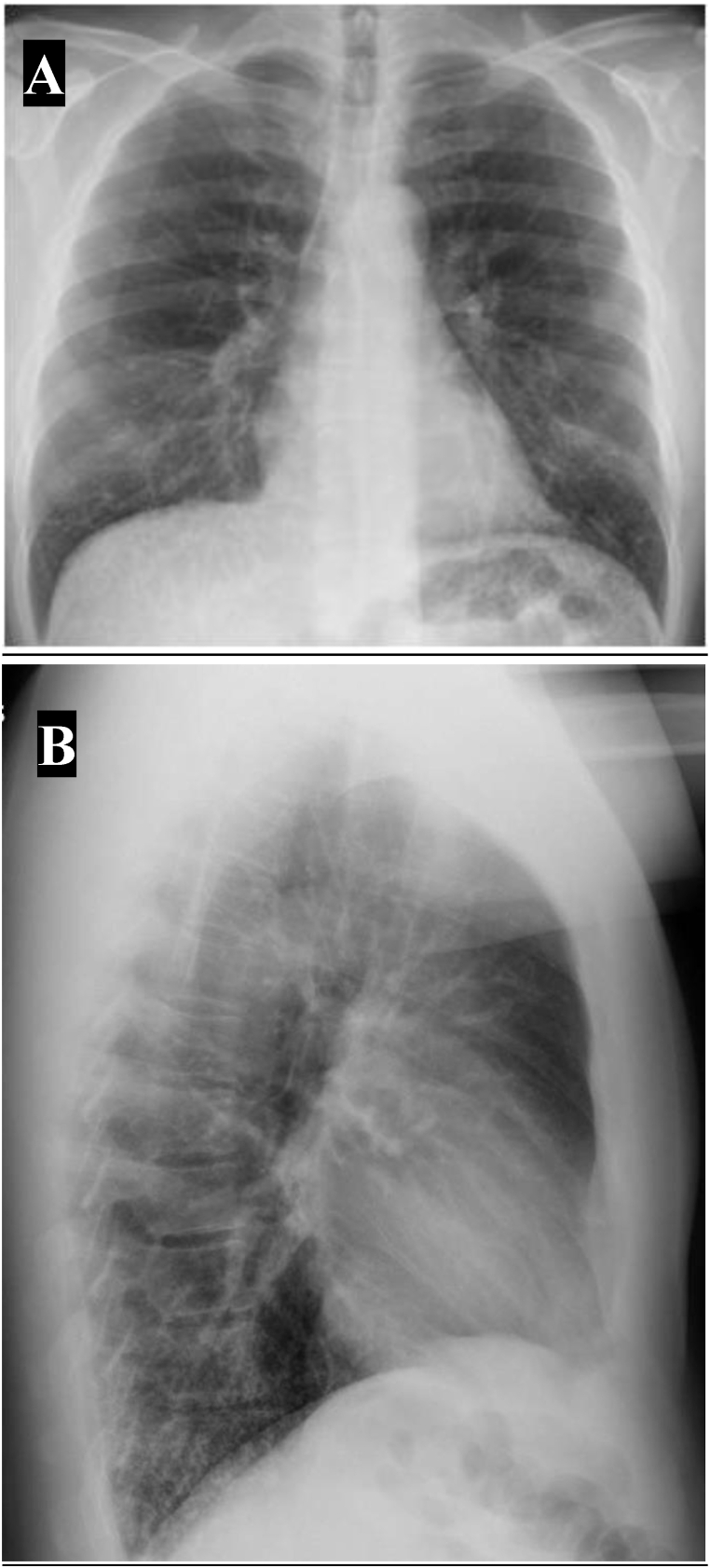
Posterior anterior (A) and lateral roentgenogram (B). Posterior basilar hyperlucent strands.

**Fig. 2:**
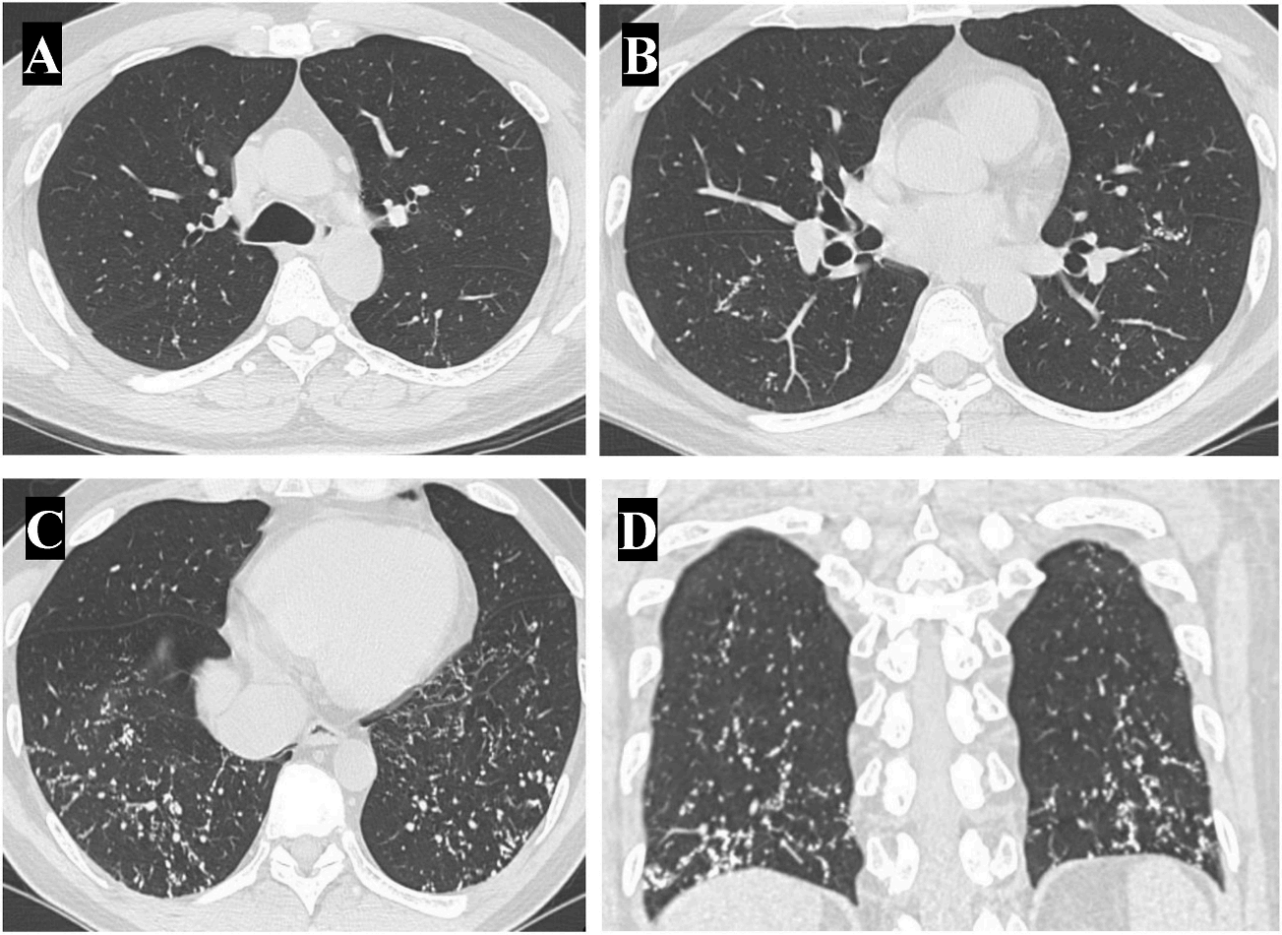
Three different computed tomography axial cuts (A–C), one coronal cut (D). Posterior basilar selective micronodular disease consistent with pulmonary ossification.

**Fig. 3:**
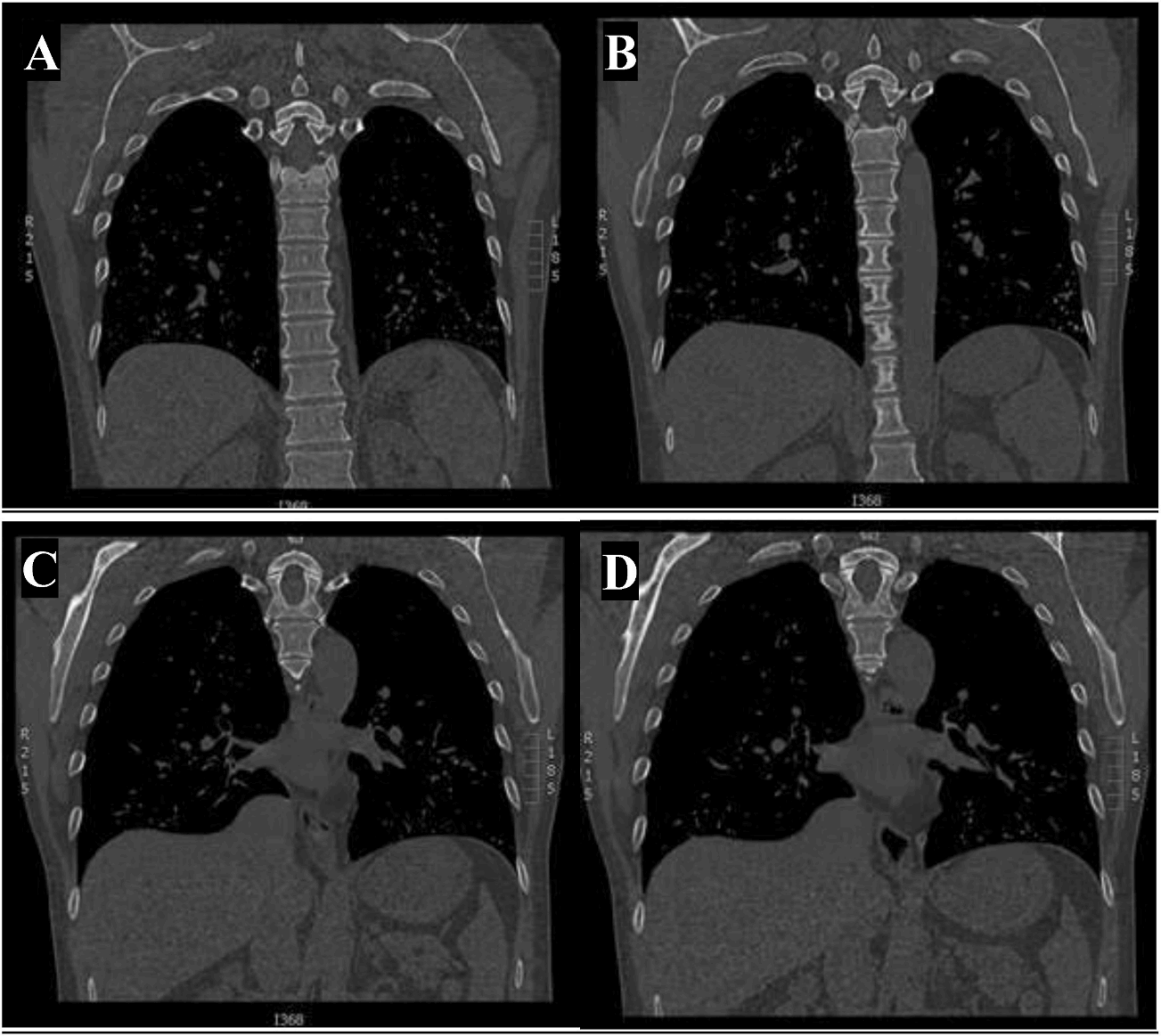
Four different computed tomography coronal cut bone windows.

Complete blood count test results acquired dating back over 5 years showed that the patient never manifested peripheral eosinophilia with the largest count of 90 eosinophils/microL. The rest of serologic studies including fungal serologies were negative. A chest roentgenogram (CXR) at the time revealed prominent reticulonodular opacities at the lung bases without significant volume loss (Fig. 1). Subsequent computed tomography (CT) of the chest demonstrated posterior basilar selective micronodular disease consistent with pulmonary ossification (Fig. 2, Fig. 3). Complete pulmonary function testing (PFT) that was performed also revealed a forced vital capacity of 6.2 L (104%), forced expiratory volume in 1 s of 4.7 L (99%), ratio of 76%, total lung capacity of 99% and a DLCO adjusted for hemoglobin of 76%.

By the time the patient was discharged to our pulmonary clinic, his viral syndrome had resolved. He denied any cough, shortness of breath, fevers, wheezing, decreased urine output, or unintentional weight loss. Given his benign asymptomatic presentation and counseling of the patient, we initially opted to monitor the patient without any initial interventions.

Approximately 1 year later, he manifested mild shortness of breath on exertion. Repeat CT of the chest demonstrated stability without any significant changes. PFTs also remained stable. Thus we offered transbronchial biopsies (TBBx) and video assisted thoracic surgery (VATS) biopsy if TBBx was inconclusive.

**Fig. 4:**
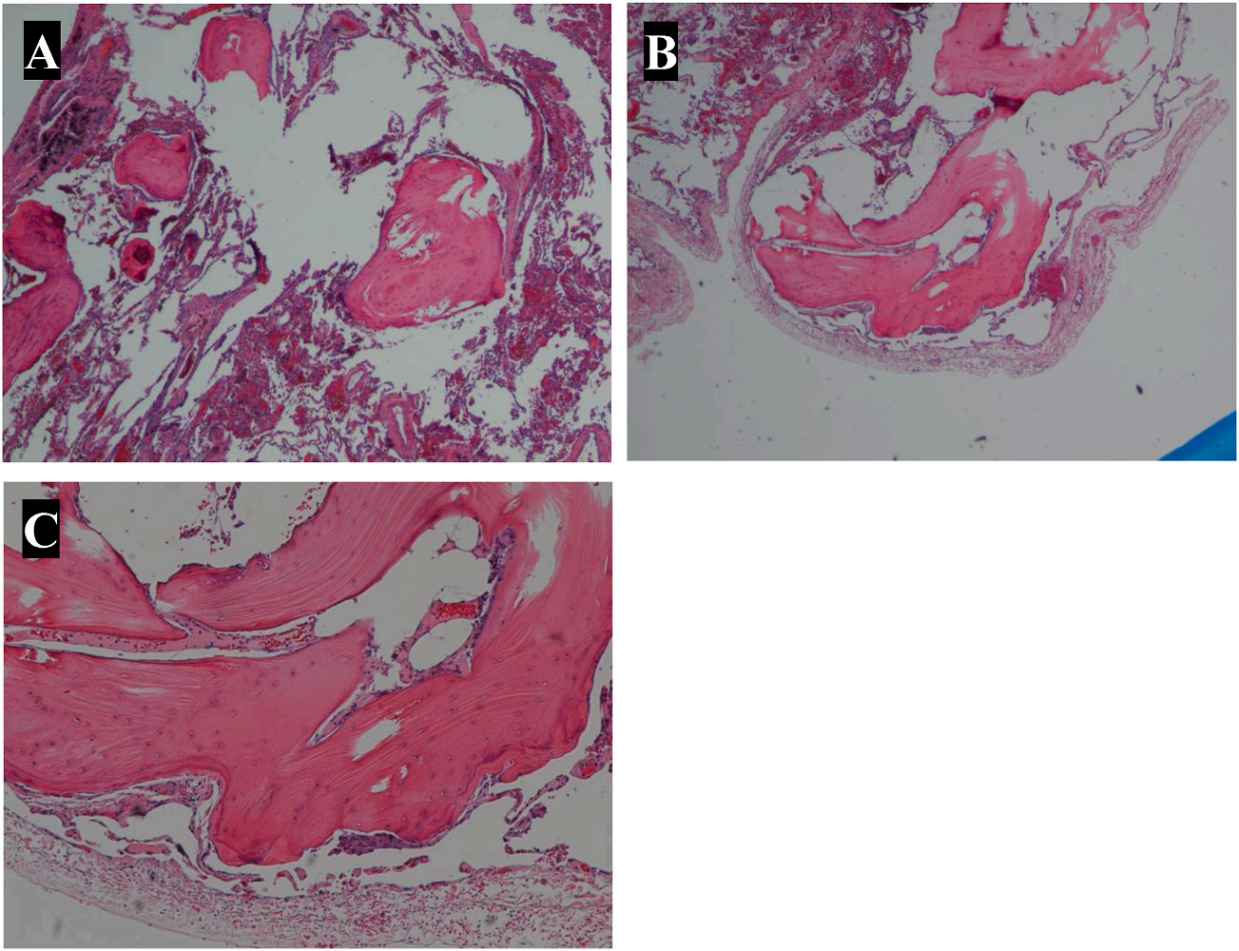
Histopathology slides showing multiple pulmonary ossifications (A), dendriform pulmonary ossification (B), and lamellar bone containing fat and hematopoietic elements (C).

Eight TBBx were taken in the right lower lobe in 4 different sub-subsegments. Pathology reported evidence of bronchial mucosa and lung parenchyma with focal interstitial fibrosis and multiple foci with dense fibrosis and calcifications, resembling osseous metaplasia. Though suggestive, ultimately non-diagnostic for PO. VATS wedge biopsies were shortly thereafter performed in the right lower lobe, right middle lobe and right upper lobe. Pathology was consistent with multifocal lobulated bone nodules with alveolar spaces with focal bone marrow consistent with dendriform pulmonary ossification (Fig. 4).

## 3 Discussion

Pulmonary ossification (PO) is an uncommon metastatic condition in which bone units form with or without marrow elements in the lung parenchyma [3]. The disease typically develops in middle-aged men between the ages of 40 and 60 years and is often asymptomatic [1,2]. However, in a minority of cases, PO manifests symptoms of an unproductive cough, chest pain, fatigue, dyspnea, and/or hemoptysis [[4], [5], [6]]. PFTs may also reveal a mild restrictive pattern with below the lower limit of normal diffusion capacity [6,7]. PO is most often identified post-mortem as a rare autopsy finding that only occurs at an incidence of 1.63 cases per 1000 autopsies in patients without any history of lung disease [8,9]. However, widespread use of radiographic imaging in pulmonary disease and technological advances in surgical biopsies have made PO a more frequent ante-mortem diagnosis.

Histologically, ossification presents in two distinct subtypes: a nodular type and a dendriform type. The dendriform type (DPO) consists of a spiculated branching pattern of bone proliferation that appears on chest radiography as linear shadows, 1–4mm thick [5]. They are often found along the alveolar septal walls and spaces and tend to include marrow elements [10]. In contrast, nodular pulmonary ossification (NPO) is characterized by lobular or round-shaped bone nodules within the alveolar spaces that are devoid of marrow elements [3,11]. Based on current literature, NPO is found more commonly than DPO [11].

NPO and DPO are thought to have different physiological processes that show overlapping patterns. Current literature has found that NPO is closely associated with chronic pulmonary congestion [3]. Specifically, NPO has been identified with cardiac valve disorders and senescent diseases such as mitral stenosis, interstitial lung disease, and idiopathic hypertrophic subaortic stenosis [[12], [13], [14]]. DPO has been attributed to chronic obstruction and inflammation. It has been seen in a myriad of conditions including histoplasmosis, chronic aspiration of gastric acid, pulmonary amyloidosis, and cystic fibrosis [1,15,16]. Even so, both NPO and DPO patterns have been observed in idiopathic cases without any underlying lung diseases [8,9,[17], [18], [19], [20]].

The mechanism of diffuse pulmonary bone formation is still unclear. One hypothesis has suggested that mutations in transcription factors regulating bone morphogenic protein (BMPs) and transforming growth factors-β (TGF-βs) stimulate ectopic bone formation in the lungs [21,22]. Another proposal by Jaderborg and Dunton speculates that a chronically anoxic and acidic environment brought upon by recurrent pneumonia and lung fibrosis promotes metaplasia of fibroblasts into osteoblasts, thus spurring bone formation in the pulmonary interstitium [8]. While these two studies have only highlighted two possible pathways of PO, it is thought that a combination of many other factors are responsible for the metastatic patterns of pulmonary ossification. Associations to PO have been documented in chronic pulmonary congestion, interstitial fibrosis, genetic markers, metallic or asbestos exposure, and angiogenesis [1,3]. However, given the low prevalence of PO, more research needs to be conducted before a specific causality can be identified.

CT has become the standard method of revealing ossification and underlying diseases in the lungs. Compared to a regular CXR, CT benefits from higher sensitivity with better resolution of parenchymal abnormalities. The presentation of NPO on CT is characterized by lobulated or round solitary nodules often resembling mucus pluggings, granulomas, or masses [23,24]. A typical differential profile for NPO includes silicotic nodules, infectious granulomas, sarcoidosis, variola pneumonias, or carcinomas [1,3,24]. In contrast, DPO on CT demonstrates a branching, coral-like, dendriform pattern that is similar to features of bronchiectasis, pulmonary fibrosis, emphysema, and lymphangitic metastasis [23,25]. More common conditions such as interstitial lung disease, amyloidosis, histoplasmosis, prior tuberculosis, or granulomatosis with polyangiitis should be considered as differential diagnoses for DPO [26]. Even with advanced imaging techniques, the radiographic differences between PO and its counterparts are still nuanced and often misdiagnosed or overlooked by many physicians.

In conjunction with a high index of suspicion, careful observation of clinical signs, and accurate interpretation of high-resolution CT, the diagnosis of PO can be confirmed with bone scintigraphy or thoracoscopic biopsy. The extraosseous accumulation of technetium-99m-methyl diphosphate (99mTc-MDP) on a bone scan is rare and often associated with abnormal calcium metabolism [27,28]. Although this pattern is seen in a handful of other malignancies and infections such as acute lymphocytic leukemia, breast cancer, and non-tuberculosis mycobacterium, the combination of 99mTc-MDP positivity with nodular or dendriform characteristic morphology in the lungs has only been observed in pulmonary ossification [[28], [29], [30]].

A minimally invasive surgical procedure known as pulmonary wedge resection by video-assisted thoracoscopic surgery (VATS) may also be performed to scope the lungs and biopsy tissue for further evaluation. Based on the pathology findings, PO would present as bone tissue formation in the lung parenchyma. The distinction between the types of PO is made depending on the shape, presence of marrow, and location of the bone units. NPO demonstrates round or lobulated bone nodules without marrow elements in the alveolar spaces [3,11,23]. DPO manifests as multiple foci of branched or tubular-shaped bone with marrow elements within the alveolar septa of the lungs [3,5,10,23].

An accurate prognosis is still unknown and difficult to determine. In most cases of PO, symptoms remain stable without progressing for many years [3]; however, a subset of patients have reported developing progressive respiratory insufficiency and *cor pulmonale* [7]. Current therapeutic approaches targeting ossification are only experimental and aim to manage symptoms or complications. Yearly CXRs and PFTs are recommended for follow-up surveillance.

## 4 Conclusion

This case study presents a rare case of dendriform pulmonary ossification (DPO) with novel pathology slides resulting from a video-assisted thoracoscopic wedge resection (VATS). We suspect the etiology of DPO in this particular patient to be due to gastroesophageal reflux disease and chronic dust exposure resulting in persistent inflammation. We have proactively treated his reflux disease, counseled him to avoid airway irritants and plan to monitor him for symptoms with a low threshold for repeat pulmonary function testing and imaging. Further recognition of this condition will enlighten treatment cascades and result in better outcomes for patients diagnosed with this enigmatic disease.

## Declaration of competing interest

Patient consents to publication.

All authors have no disclosures or conflicts of interest.

All authors were involved in the authorship of this publication.
